# Divergent impacts of quantity versus price-based monetary policies on banking systemic risk: Evidence from China

**DOI:** 10.1371/journal.pone.0322709

**Published:** 2025-05-28

**Authors:** Yichao Mo, Weihong Sun, Youqiang Ding, Li Wang

**Affiliations:** 1 Finance Department, Tongling University, Tongling, China; 2 Research Institute of Social Development, Southwestern University of Finance and Economics, Chengdu, China; Damascus University, SYRIAN ARAB REPUBLIC

## Abstract

This study investigates the effects of price-based (PMP) and quantity-based monetary policies (QMP) on systemic risks within China’s banking sector. We identify exogenous components of PMP and QMP by isolating explicit structural shocks in a structural vector autoregression model. Systemic risks are categorized into bottom-up risks, which assess how the distress or default of a single bank can contribute to systemic vulnerabilities, and top-down risks, which evaluate the likelihood of a bank experiencing distress during financial market turbulence. Utilizing a smooth local projection model, we analyze the impact of PMP and QMP on these two types of systemic risks. Our findings reveal that contractionary PMP shocks exacerbate bottom-up systemic risks while mitigating top-down risks. In contrast, contractionary QMP shocks initially elevate but subsequently diminish bottom-up risks, with minimal impact on top-down risks. Importantly, PMP affects state and non-state banks differently, decreasing top-down risks in state banks but increasing them in non-state banks. This differential impact indicates that the risk-taking behavior of non-state banks triggered by contractionary PMP can spill over, amplifying the damage of financial distress for both state and non-state banks.

## 1. Introduction

Understanding the relationship between monetary policy and systemic risk-taking in the banking sector is pivotal in the post-global financial crisis era. Monetary policy, traditionally focused on inflation control and economic stabilization, also significantly influences risk behaviors within financial institutions. As central banks worldwide adopt various monetary policy measures to stabilize economies, understanding the unintended consequences of these policies on financial stability becomes paramount. While research on the risk-taking channel of monetary policy at the individual bank level has expanded, its impact on systemic risk at the aggregate level remains underexplored [1]. Additionally, the role of monetary aggregates in influencing banking systemic risk is largely overlooked.

This paper fills these gaps by investigating how price-based (PMP) and quantity-based monetary policies (QMP) affect systemic risks in China’s banking sector. We use the SVAR methodology from Baumeister & Hamilton (B&H) [2] and Mo et al. [3] to isolate the exogenous components of PMP and QMP. The systemic risk of the banking sector is measured using seven systemic risk indicators. Referring to Drehmann [4], these indicators are categorized into five bottom-up measures, which evaluate how a single bank’s distress or default can contribute to systemic vulnerabilities, and two top-down measures, which evaluate risk from a systemic perspective and distribute it across individual institutions. We synthesize these measures into two composite indicators—SR1 for bottom-up risks and SR2 for top-down risks—using principal component analysis (PCA). Finally, we apply a smooth local projection (SLP) model to evaluate the impact of PMP and QMP shocks on SR1 and SR2.

China is an intriguing case study for three reasons. First, unlike developed economies that primarily use interest rates, China frequently employs both price-based and quantity-based monetary tools, such as interest rates and reserve requirement ratios [5,6]. This dual approach provides a unique opportunity to examine the impact of monetary policy on systemic risk, offering insights relevant to advanced economies where non-price-based policies like QE and LSAP remain crucial. Second, China’s financial system is predominantly bank-driven, especially by large State banks [7], contrasting with market-based systems of countries like the U.S.. These institutional characteristics offer a natural environment for exploring how monetary policy influences banking systemic risk. Third, China’s ongoing property market downturn and economic slowdown, worsened by the COVID-19 pandemic, pose huge risks to China’s banking sector, which is heavily exposed to real estate. Understanding the influence of Chinese monetary policy on systemic risk is essential for economic stability, guiding effective policy reform, and mitigating potential global financial repercussions.

Three main findings emerge from our analysis, revealing distinct impacts of PMP and QMP on China’s banking systemic risks. First, contractionary PMP shocks increase bottom-up systemic risks (SR1) while decreasing top-down systemic risks (SR2). In contrast, contractionary QMP shocks initially raise SR1 but ultimately reduce it, with no significant effect on SR2. This finding underscores the importance for policymakers, particularly in emerging economies, to select these policies based on the specific systemic risks they aim to contain. For example, to gradually reduce SR1 without impacting SR2, contractionary QMP might be preferable.

Second, when examining the role of bank ownership, the effects of QMP on SR1 and SR2 remain consistent across both state and non-state banks. However, a tightening PMP increases SR1 in both state and non-state banks. It reduces SR2 in state banks but increases SR2 in non-state banks. This suggests that the risky behavior of non-state banks, triggered by contractionary PMP, could have spillover effects on the entire financial system. Therefore, a cautious approach to raising interest rates is essential, potentially supported by targeted measures to assist non-state banks in mitigating systemic risks. For example, macroprudential policies could be adjusted to provide additional buffers for non-state banks during periods of monetary tightening.

Third, bank size and funding structure also influence the impacts of monetary policy on banking systemic risk. Specifically, larger asset size enhances the effects of both PMP and QMP on SR1. A lower dependency on deposit funding increases the positive impact of contractionary PMP on SR2 but decreases it for contractionary QMP. This set of results underscores the importance of coordinating monetary and macroprudential policies. For example, macroprudential tools like countercyclical capital buffers could be adjusted based on the asset size and non-deposit liability of banks to manage systemic risks more effectively.

Our study mainly contributes to two strands of literature. The first strand focuses on systemic risk and its measurement, encompassing various methodologies and indicators [8]. Systemic risk refers to the potential for a disturbance at financial institutions or financial markets to trigger widespread instability, adversely affecting the real economy. This risk arises from the interconnectedness of financial institutions, contagion potential, and macroeconomic shocks. Drehmann [4] classifies systemic risk indicators into bottom-up and top-down approaches. The bottom-up analysis focuses on micro-level interactions contributing to systemic vulnerabilities, using methods such as Catastrophic Financial Risk (CATFIN; [9]) and Delta CoVaR (△CoVaR, [10]). The top-down measures, like systemic expected shortfall (SES, [11]) and SRISK [12], assess system-level risks in the financial system and their distribution across institutions. These indicators have demonstrated predictive capabilities during financial crises and economic downturns in advanced economies [9–11]. Our study employs both perspectives to assess the banking systemic risk in China, revealing that while China’s bottom-up systemic risk remains minimal, its top-down systemic risk has significantly escalated, reaching unprecedented levels after 2022.

The second strand examines the impact of monetary policy on systemic risks in the banking sector. Many studies have shown that expansionary PMP prompts riskier behavior in banking sector by using either panel data or macro time series [13–20]. In contrast, Chen et al. [21] find that in China, it is the contractionary monetary policy rather than the expansionary that increases risk-taking among non-state banks. Beyond the discussion on PMP, some studies have explored how unconventional monetary policies affect systemic risks, finding that while quantity easing (QE) or large-scale asset purchases (LSAP) increases risk-taking in the banking sector, they can lower the systemic risks overall [13,22–24]. Although numerous studies examine the impact of monetary policy on systemic risks, few have discussed the impact of QMP on systemic risks (e.g., [25]), despite the QMP is still important in developing countries, particularly in China. Given China’s ongoing housing price slump and monetary policy expansion to counter the economic downturn, it is crucial to discuss in detail how China’s monetary policy affects systemic risks. Our study adds to this literature by further examining the impact of QMP and demonstrating that PMP and QMP have divergent effects on systemic risks.

The remainder of this paper proceeds as follows: Section 2 provides the institutional background regarding China’s monetary policy framework and banking system. Section 3 explores the impacts of China’s PMP and QMP on its banking systemic risks. Section 4 offers robustness checks. Finally, Section 5 concludes the paper.

## 2. Institutional background of China’s monetary policy and banking system

In this section, we discuss the institutional aspects of China’s monetary policy framework and banking system that are essential for our forthcoming empirical analysis. Our discussion initiates with an overview of China’s monetary policy framework, proceeds with an examination of its banking system, and concludes by analyzing the relationship between China’s monetary policy framework and its systemic banking risks.

### 2.1. China’s monetary policy framework

The current monetary policy framework in China is characterized by its state-controlled and coordinated approach, multiple policy objectives, and a mix of quantity-based and price-based instruments [5]. First, China’s central bank—the People’s Bank of China (PBoC) — operates under the leadership of the State Council, which means that monetary policy decisions are highly coordinated with broader government policies. This centralization contrasts with the more independent central banks in advanced economies. Second, the primary objectives of China’s monetary policy include maintaining price stability and promoting economic growth [26]. The People’s Bank of China (PBoC) also focuses on managing financial stability and supporting employment. In contrast to the US economy, where the primary focus of monetary policy is on controlling inflation, China, as an emerging market economy, prioritizes the central government’s aim of meeting its annual GDP growth target. Third, the PBoC historically has relied heavily on quantity-based instruments such as reserve requirements and direct credit controls [27]. These instruments are used to regulate M2 growth, which officially served as the intermediate target from 2000 to 2018 to bolster GDP growth while maintaining inflation stability. In recent years, there has been a shift towards greater use of price-based instruments, such as interest rates [28].

#### 2.1.1. Price-based monetary policy.

Between 1996 and 2015, China embarked on a gradual path to deregulate interest rates. This process of interest rate liberalization began with interbank rates. In 1996 and 1997, rates in the interbank money market, like CHIBOR and repo rates, were deregulated. By 2005, interbank deposit rates followed suit. Meanwhile, the PBoC gradually widened the allowable range for benchmark lending and deposit rates. Significant steps were taken in October 2004 when the upper limit on lending rates and the lower limit on deposit rates were removed. Further advancements occurred in July 2013 and October 2015, with the removal of the lending rate floor and the deposit rate ceiling, respectively, thus completing the liberalization process for retail lending and deposits.

This progress led the PBoC to adopt the interbank rates as key monetary policy targets. The Shanghai Interbank Offered Rate (SHIBOR) was established in 2007 to serve as a benchmark, much like LIBOR, calculated from the daily offered rates of participating banks. However, SHIBOR’s impact was limited as it reflected quoted prices rather than actual transactions. In 2015, the PBoC shifted to using the 7-day reserve repo rate (R007) as its primary policy target. The market now regards both the R007 and the three-month SHIBOR rate as critical benchmarks for other financial instruments. For the empirical analysis below, we employ the R007 as the primary short-term price-based policy instrument.

Before August 2019, the most important rates for commercial loans and deposits were the benchmark lending and deposit rates. The PBoC developed a market-based Loan Prime Rate (LPR) for banks to price credit risk, and by August 2019, it mandated that all new loans should use the LPR for pricing. Despite the total liberalization of interbank rates, the deregulation of RMB lending and deposit rates remains incomplete. To curb rate competition, major banks in China introduced a self-regulatory mechanism in October 2015, capping the upward floating ratio of deposit rates at 50% of the benchmark [5]. The 2016 Macroprudential Assessment System further regulated deposit rate pricing to prevent competitive escalation. Hence, China maintains a dual-track interest rate system: while interbank rates are fully deregulated, a de facto ceiling on bank deposit rates persists.

To summarize, the PBoC is gradually transitioning from a quantity-based to an interest rate-based monetary policy framework, supported by the liberalization of interest rates. At present, both PMP and QMP instruments are actively employed in China to ensure macroeconomic stability.

#### 2.1.2. Quantity-based monetary policy.

From 1984 to 1997, China’s monetary policy concentrated on regulating bank credit. This changed on January 1, 1998, when the PBoC switched its monetary policy from controlling bank credit to supervising annual broad money supply (M2) growth. Key decisions regarding quarterly adjustments to M2 growth are made by the Politburo, which includes the General Secretary of the Chinese Communist Party, the Premier of the State Council, and other high-ranking central government officials, including the Governor of the PboC. Unlike the Federal Reserve System, the PBoC does not operate independently from other central government units. In practice, the PBoC adjusts M2 growth rates quarterly in reaction to economic conditions while ensuring these changes align with the annual M2 growth targets established by the State Council. From 2000 to 2018 [21].

The PBoC employs a variety of instruments to achieve the M2 growth target, including open market operations, the central bank base interest rate, central bank lending, reserve requirements, rediscounting, and other tools specified by the State Council. Here, we focus on two major instruments: open market operations and reserve requirement changes. Established in May 1998, the open market operations system has developed significantly over the past three decades, becoming the regular tool for the PBoC to regulate the money supply. Bond trading through open market operations encompasses spot trading, repurchase agreements, and the issuance of central bank bills and government bonds. Repurchase agreements are categorized into repo and reverse repo. The reserve requirement system, initiated in 1984, uses changes in the reserve requirement ratio (RRR) to assist in meeting the M2 growth target, though it is used less frequently than open market operations.

### 2.2. Banking system

A distinctive trait of China’s banking system is the dominance of large state banks. There are six major state banks directly overseen by the central government: Industrial and Commercial Bank of China, China Construction Bank, Bank of China, Agricultural Bank of China, Bank of Communications, and Postal Savings Bank of China. On the other hand, the non-state banking sector comprises joint stock banks, city commercial banks, and rural commercial banks, collectively accounting for nearly half of the system’s overall size. To illustrate, in 2022, their assets made up 43.8 percent of the total banking system.

Two institutional distinctions between state and non-state banks stand out. First, their funding sources differ notably. state banks operate a nationwide network of branches across all provinces in China, while non-state banks typically have a localized presence, such as city or rural commercial banks, or maintain a limited number of branches outside their headquarters’ province [29]. Consequently, non-state banks face considerable challenges in attracting deposits compared to state banks. This challenge is further intensified by a deposit rate ceiling, whether outright or implied, making it difficult for non-state banks to increase their deposit base by offering substantially higher deposit rates than state banks. This disparity is evident when examining the composition of total liabilities: in 2022, deposits accounted for 79.92% of total liabilities for state banks, compared to just 65.94% for publicly listed non-state banks.

Second, non-state banks exhibit a greater risk-taking propensity compared to their state counterparts. This difference stems from various factors associated with their ownership structure, the regulatory framework, and the pressures of competing in the market. Non-state banks are primarily motivated by the pursuit of profits and the need to navigate competitive forces, often leading them to engage in higher-risk activities for potentially greater returns. They generally benefit from more independent governance structures, allowing them to make decisions without direct government oversight. This autonomy can pave the way for more assertive risk-taking strategies due to the absence of stringent governmental control.

### 2.3. Monetary policy and banking systemic risks

Before presenting the formal empirical analysis, it is helpful to review several episodes to illustrate how QMP and PMP have affected banking systemic risk in recent years.

#### 2.3.1. PMP and banking systemic risks.

PMP affects state and non-state banks in China differently due to variations in their funding structures, risk-taking behaviors, and roles in the financial system. State banks benefit from a more elastic deposit supply due to their nationwide network of branches and government backing, which allows them to maintain a stable credit supply and are less affected by changes in the cost of funding when policy interest rates are adjusted. This stability reduces their need to engage in risky lending practices and enables them to act as net suppliers in the wholesale funding market. In contrast, non-state banks experience more volatility in their credit supply due to their dependence on wholesale funding and less elastic deposit base. This volatility can lead to periods of rapid credit expansion followed by sharp contractions, increasing systemic risk. While wholesale funding via interbank certificates of deposit boosts loan supply from non-state banks during monetary policy easing in China, it concurrently heightens interconnectedness within the banking system and non-state banks’ leverage. This, in turn, elevates systemic financial risks, particularly in the face of adverse economic events such as trade wars and the Covid-19 crisis.

**Fig 1:**
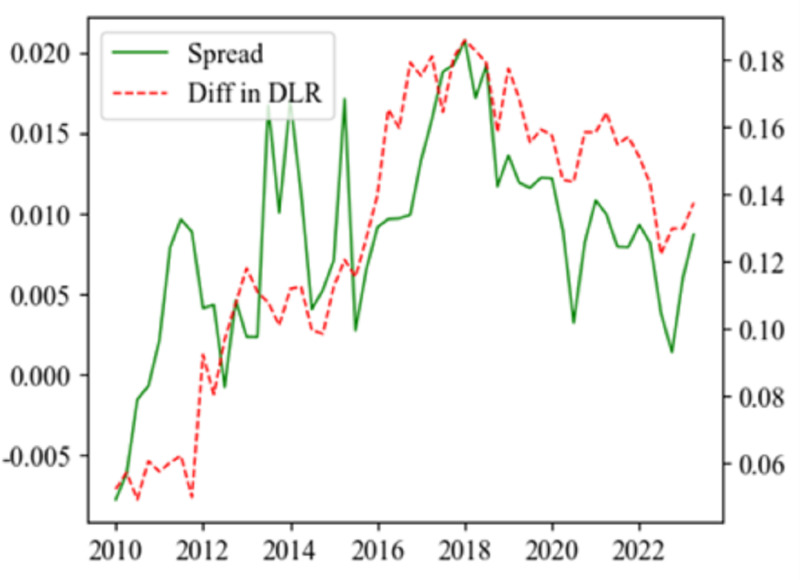
R007 and the difference in deposit liability ratio. Notes: The solid line denotes the spread between R007 and deposit interest rates, while the dashed line shows the difference in deposit liability ratios between state banks and non-state banks.

Non-state banks, which rely heavily on wholesale funding and riskier investments, face heightened challenges and systemic risks during periods of monetary tightening. When PMP started to tighten in 2009:II, the R007 rose from 1% to 3.42% by 2018:II. Under the dual-track system, this rise led to significantly higher costs for interbank liabilities costs than deposit liabilities. Consequently, state banks boosted their deposit liabilities, pulling capital resources from non-state banks. Fig 1 illustrates that the spread between R007 and deposit interest rates widened from 2010 to 2018, mirroring the growing disparity in deposit liability ratios between state and non-state banks. After 2018, as this spread narrowed, the difference in deposit liability ratios similarly decreased. This shift forced non-state banks to depend more on expensive interbank liabilities, squeezing their profit margins and increasing financial stress, particularly for smaller and less established ones. To maintain profitability, non-state banks engage in riskier lending practices such as extending credit to higher-risk borrowers or investing in higher-yield but riskier assets through shadow bank activities [21]. This behavior can increase their exposure to both bottom-up and top-down systemic risks.

**Fig 2:**
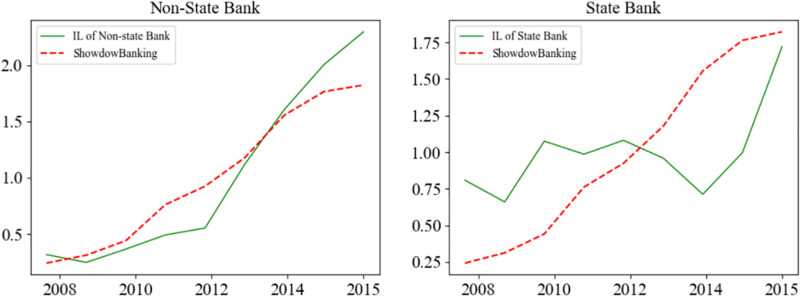
The scale of interbank liability and shadow banking loan. Notes: The solid line illustrates the volume of interbank liabilities, and the dashed line marks the volume of shadow bank loans; values are adjusted to be mean-centered. Data originates from CEIC and Chang et al. [30].

Meanwhile, contractionary PMP in China from 2009 to 2015 correlates positively with the increase in non-state banks’ interbank liability and shadow banking’s scale. During the period of monetary contraction, funding costs for non-state banks escalate, while loan interest rates remain strictly regulated. This situation markedly diminishes the profitability of non-state banks. As a response, these banks sidestep regulatory constraints by delving into shadow banking activities. Fig 2 demonstrates a close correlation between the expansion of shadow banking and an increase in interbank liabilities for non-state banks, a pattern that is not mirrored in state banks. Shadow banking, known for its high-risk and high-return profile, appeals to borrowers who face challenges in obtaining conventional loans and are therefore willing to accept higher interest rates. This borrower demographic often includes State enterprises (SOEs) from financially beleaguered regions, high-risk real estate developers, and sectors plagued by overproduction. By allocating funds to shadow banking, non-state banks not only expose themselves to an elevated risk of financial distress but also play a role in deteriorating the financial system’s stability.

#### 2.3.2. QMP and banking systemic risk.

**Fig 3:**
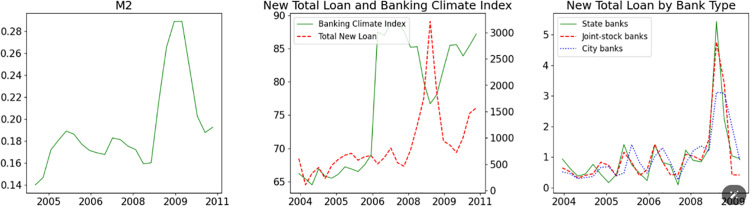
China’s M2 growth, total new loan, and Banking Climate Index. Notes: The left panel displays the M2 growth rate. In the middle panel, the solid line represents total new loans, while the dashed line depicts the Banking Climate Index. The right panel shows the total new loans provided by different banks; each series is normalized by its mean. Data sourced from Chang et al. [30] and CEIC.

While expansionary QMP can quickly stimulate the economy, it often leads to financial resources being channeled into inefficient investments, thereby undermining China’s economic stability and exacerbating the impact of banks’ distress on the financial market in the long term. During periods of QMP loosening, banks receive increased liquidity from the PBoC and subsequently expand their loan portfolios. Fig 3 illustrates a significant rise in M2 in the first quarters of 2009 in response to the 2008 Global Financial Crisis, followed by notable expansions in new total loans. However, the Banking Climate Index notably declined during the same period, indicating that the increase in loans was driven more by governmental directives than market dynamics.

**Fig 4:**
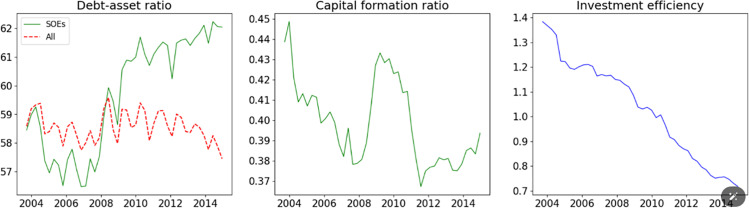
Debt and Investment of SOE. Notes: The debt-to-asset ratio is the proportion of a firm’s total liabilities to its total assets. The capital formation ratio represents the share of SOE capital formation in the total capital formation. Investment efficiency is measured as the ratio of SOE capital formation to SOE fixed asset investment.

The 4 trillion yuan stimulus package announced in 2008 was accompanied by significant monetary policy measures, including reductions in interest rates and reserve requirements. These extraordinary actions led to a significant surge in bank lending during 2008–2009, primarily directed toward SOEs, resulting in a rapid increase in their investments [31]. Fig 4 illustrates that the debt-asset ratio of SOEs dramatically rose during this period, while the debt-asset ratio for all enterprises remained stable, indicating that the increased lending was predominantly aimed at SOEs. With this new bank lending and under government guidance, SOEs swiftly escalated their investments. The proportion of SOEs’ gross fixed capital formation (GFCF) in total GFCF sharply rose from 2008 to 2010. However, much of this increased investment by SOEs was channeled into land acquisitions and overinvestment in unprofitable infrastructure projects. Following this investment spike, the investment efficiency of SOEs consistently declined after Q3 2009, eventually leading to a debt overhang among local governments and State enterprises, thus threatening financial stability.

## 3. Empirical analyses

### 3.3. Empirical methodology

#### 3.3.1. Data.

Table 1 provides a summary of our series name, data source, data frequency, and period. To identify PMP and QMP shocks, we utilize data from CEIC to establish a four-variable SVAR, which includes real GDP, the GDP deflator, M2, and R007. This dataset spans from 1996: I to 2023: I. The selection of these variables is well-established in the literature for identifying monetary policy shocks (e.g., [32,33]). Notably, the order of variables in the SVAR does not affect our empirical results.

**Table 1:** Data Summarization.

	Series	Source	Frequency	Span
Identifying PMP and QMP shocks	Real GDP	CEIC	Quarterly	1996: I - 2023: I
	GDP deflator	CEIC	Quarterly	1996: I - 2023: I
	M2	CEIC	Quarterly	1996: I - 2023: I
	R007	CEIC	Quarterly	1996: I - 2023: I
Calculating systemic indicators	Stock Prices	CSMAR	Daily	2007:I -2023:I
	Trading Volumes	CSMAR	Daily	2007:I -2023:I
	Market Capitalizations	CSMAR	Daily	2007:I -2023:I
	Real Estate Stock Index	CSMAR	Daily	2007:I -2023:I
	CSI 300 Index	CSMAR	Daily	2007:I -2023:I
	VIX Index	-Calculated based on CSI 300 Index	Daily	2007:I -2023:I
	R007	CEIC	Daily	2007:I -2023:I
	3-month Government Bond Yield	CEIC	Daily	2007:I -2023:I
	10-year Government Bond Yield	CEIC	Daily	2007:I -2023:I
	Total bank Assets	CSMAR	Quarterly	2007:I -2023:I
	Total bank Equity	CSMAR	Quarterly	2007:I -2023:I

To calculate the systemic indicators of the banking sector, we require both macroeconomic series and micro-level bank financial data. The data covers the period from 2007:I to 2023:I, as most listed bank financial series are unavailable before 2007. The macroeconomic series, sourced from CEIC, includes daily R007, daily 3-month government bond rate, and daily 10-year government bond rate. The microdata is obtained from the China Stock Market & Accounting Research Database (CSMAR), encompassing daily stock prices, daily trading volumes, and market capitalizations for 4 state banks and 10 non-state banks, and daily real estate stock index, daily CSI 300 index. Additionally, we incorporate quarterly total assets and equity for each bank. The daily VIX index is the standard deviation of the CSI 300 index over 22 days.

#### 3.3.2. Identification scheme for PMP and QMP shocks.

To study the effects of PMP and QMP, we must address two challenges: the endogeneity of monetary policy with the macroeconomy, and the interdependence between the targets of PMP and QMP, namely M2 and R007. We tackle these challenges by modeling an SVAR using the identification method by B&H and an identification scheme by Mo et al. [3]. Instead of identifying reduced-form shocks, the method by B&H can estimate the structural shock that directly measures the exogenous component of monetary policy. Particularly, B&H’s method enables us to manually set the structural equations and prior structural parameters based on theoretical research. This flexibility allows us to define separate structural equations for PMP and QMP, thereby distinguishing between the two types of monetary policy shocks. Our structural equations and prior structural parameters are consistent with Mo et al. [3], and contemporaneous equations can be summarized as equations (1) through (4).


$$y_{t}=k^{s}+\alpha^{s}\pi_{t}+{\tilde{u}}_{t}^{s},$$ (1)

$$y_{t}=k^{d}+\beta^{d}\pi_{t}+\beta^{r}i_{t}+\beta^{m}m_{t}+{\tilde{u}}_{t}^{d},$$ (2)

$$\mathrm{\backslash [}i_{t}=c^{i}+\rho_{r}i_{t-1}+\left(1-\rho_{r}\right)\psi^{\pi }\pi_{t}+\left(1-\rho_{r}\right)\psi^{y}y_{t}+\varepsilon_{t}^{r}\mathrm{\backslash ]}$$ (3)

$$\mathrm{\backslash [}m_{t}=c^{m}+\rho_{m}m_{t-1}+\left(1-\rho_{m}\right)\gamma^{\pi }\pi_{t}+\left(1-\rho_{m}\right)\gamma^{y}y_{t}+\varepsilon_{t}^{m}.\mathrm{\backslash ]}$$ (4)

(1) and (2) are Phillips curve and IS curve, where *y*_*t*_ is real GDP growth, *π*_*t*_ is inflation, *i*_*t*_ is nominal policy interest rate, *m*_*t*_ is nominal money growth rate, *ũ*_*t*_^*s*^ is supply shock, *ũ*_*t*_^*d*^ is demand shock. (3) and (4) are PMP and QMP equations, where ***ε***_*t*_^*r*^ is PMP shocks and ***ε***_*t*_^*m*^ is QMP shocks.

We further supplement the identification with three reasonable sign restrictions depicted by ***H***_***1***_***-H***_***3***_ to further distinguish different structural shocks. For each sign restriction, we also allow a 7.1% chance that the restriction is invalid.

***H1:*** a positive supply shock increases the contemporaneous output.

***H2:*** a contractionary PMP shock raising the interest rate has a contemporaneous negative impact on the output.

***H3:*** a contractionary QMP shock decreasing the money supply has a contemporaneous negative impact on the output.

#### 3.3.3. Systemic risk indicators.

Many systemic risk indicators were designed to measure systemic risks following the 2008 Global Financial Crisis. Prevalent examples include the ∆CoVaR and the SRISK. Although each of these indicators is meticulously designed, there is concern about whether a single indicator is sufficient to capture systemic risks. Indicators such as MES and ∆CoVaR are criticized for primarily reflecting the market risk, leading to confusion between systematic risks and systemic risks, a problem less pronounced for SRISK [8]. However, Greenwood [34] pointed out that market risk is strongly associated with a higher probability of a financial crisis. Drehmann [4] and Zhou et al. [35] noted that systemic risks can be divided into bottom-up systemic risks and top-down systemic risks. These discussions indicate that a single indicator is not enough to capture all facets of systemic risks.

To mitigate the worry that a single indicator is insufficient to measure systemic risks, our study considers the seven popular systemic risk indicators, including Systemic Expected Shortfall (SES), Marginal Expected Shortfall (MES), SRISK, Catastrophic Financial Risk (CATFIN), Conditional Autoregressive Value at Risk (CAViaR), Conditional Value at Risk (CoVaR) and △CoVaR. These indicators are summarized in Table 2.

**Table 2:** Source of Systemic Risk Indicators.

Systemic risk indicators	Source
SES	Acharya et al. [11]
MES	Acharya et al. [11]
SRISK	Brownlees & Engle [12]
CATFIN	Allen et al. [9]
CAViaR	White et al. [36]
CoVaR	Adrian & Brunnermeier [10]
△CoVaR	Adrian & Brunnermeier [10]

#### 3.3.4. Baseline model.

To estimate the effects of PMP and QMP shocks on systemic risks, we employ the smooth local projections (SLP) method introduced by Barnichon and Brownlees [37]. We do not use SVAR directly for this analysis, as doing so would implicitly assume a structural relationship between monetary policy shocks and systemic risk indicators [2,39]. In contrast, LP does not make such assumptions, offering greater flexibility in model specification. This flexibility further allows for the seamless integration of interaction terms, enabling a more comprehensive analysis of the factors shaping the impacts of PMP and QMP on SR1 and SR2. SLP is an extension of the local projection (LP) method developed by Jordá [38], retaining the advantages of LP while improving estimation accuracy.

Our baseline model can be specified as follows:


$$\begin{array}{c} SR_{t+h}=\alpha_{h}+\beta_{h}MP_{t}+\gamma_{1}SR_{t-1}+\gamma_{2}SR_{t-2}\\ +\eta_{1,\;h}{GDP}_{t}+\eta_{2,\;h}{GDP}_{t-1}+\eta_{3,\;h}{GDP}_{t-2}+u_{t+h} \end{array}$$ (5)

where *h* is prediction period ranging from 0 to 20, *SR* denotes SR1 or SR2, *MP* is PMP or QMP shocks, GDP is GDP real growth rate, *u* is the prediction error. Following Barnichon and Brownlees [37], we shrink the estimated IR toward a line by setting the estimated multiplier *β*_*h*_ toward to a linear polynomial, which is roughly consistent with the IR estimated by the standard LP.

### 3.4. Baseline results

#### 3.4.1. PMP and QMP shocks.

**Fig 5:**
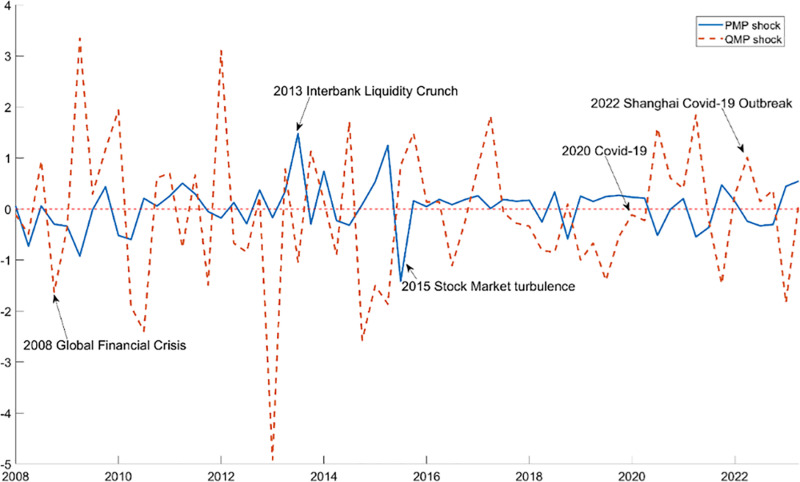
China’s PMP and QMP Shocks. Notes: The solid and dashed lines represent the shocks from China’s PMP and QMP respectively, with PMP targeting the short-term interest rate and QMP focusing on the M2 growth rate.

Fig 5 displays the identified shocks for China’s PMP and QMP, spanning from 2008:I to 2023:IV. The solid line represents PMP shocks and the dashed line shows QMP shocks. Both types of shocks varied sharply following major economic events such as the Global Financial Crisis in 2008, the turbulence in the China stock market in 2015, and the outbreak of the COVID-19 pandemic in 2020. The most acute PMP shock occurred in 2013:II when the PBoC curtailed liquidity infusions into the interbank market to curb shadow banking expansion. Conversely, the largest QMP shock happened during the 2008 global financial crisis, marked by a rapid rise in China’s M2 growth rate from 16% in 2008:IV to 26.6% in 2009:II. Overall, the fluctuations in PMP and QMP shocks illustrated in Fig 5 closely correlate with shifts in China’s economic landscape.

#### 3.4.2. Systemic risk indicators.

Table 3 presents correlations between different systemic risk measures, all at a 1% significant confidence level. It clearly shows that the systemic risk measures can be divided into two groups based on their correlation. One group includes CATFIN, CAViaR, CoVaR, △CoVaR, and MES, while another contains SES and SRISK, with high positive correlations within each group. Referring to Drehmann [4] and Zhou et al. [35], the group containing △CoVaR reflects bottom-up systemic risk, while the group containing SRISK presents top-down systemic risk. Furthermore, the indicators between the two groups tend to be negatively correlated, indicating a negative correlation between bottom-up and top-down systemic risk in China. The negative correlation may be attributed to the Chinese government’s tendency to strengthen regulation or rescue financially distressed banks.

**Table 3:** Correlations between different systemic risk measures.

	CAViaR	CoVaR	△CoVaR	MES	CATFIN	SRISK	SES
CAViaR	1.00						
CoVaR	0.91	1.00					
△CoVaR	0.95	0.91	1.00				
MES	0.94	0.89	0.96	1.00			
CATFIN	0.70	0.58	0.62	0.68	1.00		
SRISK	-0.42	-0.35	-0.35	-0.39	-0.58	1.00	
SES	-0.40	-0.33	-0.36	-0.40	-0.50	0.93	1.00

Notes: This table describes the correlation between different risk measures, with all correlation coefficients significant at a 1% confidence level.

**Fig 6:**
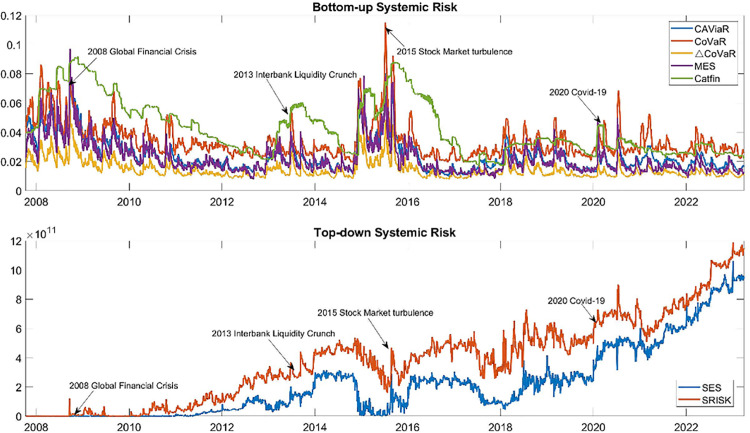
Systemic risk indicators. Note: The figure differentiates two types of systemic risk indicators: bottom-up indicators, assessing the impact of bank distress on the financial market, and top-down indicators, evaluating the likelihood of a bank experiencing distress during financial market turbulence.

Fig 6 depicts two types of systemic risk measures. The upper panel illustrates indicators of bottom-up systemic risk, which signifies the potential damage from distressed banks to the entire financial system. Most bottom-up systemic risk indicators exhibit four peaks, occurring in the 2008 Global Financial Crisis, the 2013 interbank liquidity crunch, the 2015 China stock market crash, and the 2020 Covid-19 pandemic. This suggests that bank distress could lead to greater damage during periods of financial market turmoil.

The lower panel describes the indicators of top-down systemic risk, which signify the likelihood of a bank falling into distress during financial turbulence. The value of top-down systemic risk was unusually low during the 2008 global financial crisis, indicating the strong robustness of China’s banking system at that time. This resilience could be attributed to China’s strict capital controls, which limited Chinese banks from investing in the international financial market. The value of top-down systemic risk increased rapidly and reached a historic high after 2022, coinciding with a decrease in China’s housing prices and sales. The surge in top-down systemic risk suggests an increasing likelihood for China that financial market turbulence could evolve into a financial crisis.

To efficiently utilize the information from different systemic risk indicators and avoid redundancy, we perform Principal Component Analysis (PCA) on both bottom-up and top-down systemic risk indicators. Before conducting PCA, non-stationary series are first-order differenced to obtain stable series, and all stationary series are standardized. Table 4 displays the variance-explained ratio of a single component. It is observed that both bottom-up and top-down systemic risk indicators can be effectively summarized with one component, each explaining over 80% of the variance. The PCA components derived from bottom-up and top-down risks are denoted as SR1 and SR2, respectively, and measure two kinds of systemic risk in our analysis.

**Table 4:** PCA for Systemic Risk Measures.

Group	Components	Variance Explained Ratio
Bottom-up systemic risk indicators	1	89.60%
Top-down systemic risk indicators	1	80.89%

Notes: Bottom-up systemic risk indicators measure potential damage from a bank’s distress to the financial system, while top-down risk indicators assess the likelihood of a bank falling into distress during financial market turbulence.

#### 3.4.3. The effects of PMP and QMP on systemic risks.

**Fig 7:**
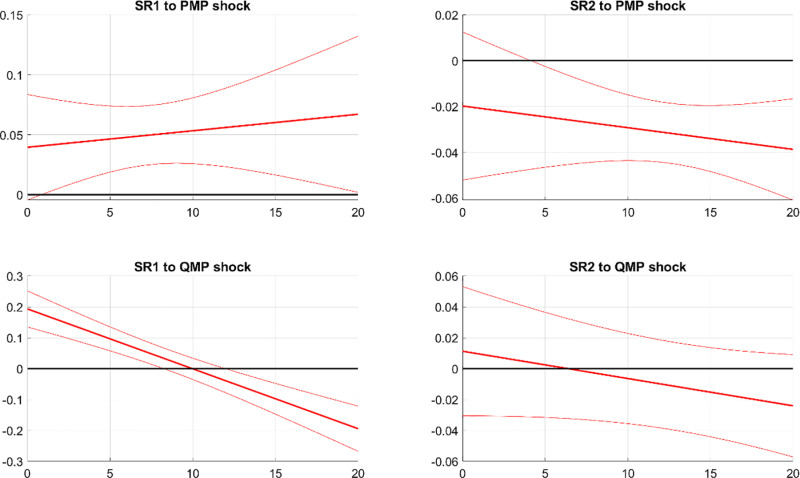
IRFs of banks’ systemic risks to PMP and QMP shocks. Notes: The figure illustrates the impact of contractionary PMP and QMP shocks on banking systemic risks. SR1 and SR2 represent the potential damage from a bank’s distress to the financial system and the likelihood of distress during market turbulence respectively. The solid line shows the median response, and the dashed bands indicate the 68% confidence interval.

Fig 7 demonstrates that PMP and QMP shocks differ in their effects on the systemic risk of the banking sector. The first column of Fig 7 illustrates that the impulse response functions (IRFs) of SR1 are significantly positive following a contractionary PMP shock. This finding aligns with previous research indicating that unexpected monetary policy tightening can increase systemic risk rather than reduce it [21,40,41]. By expanding shadow bank activities, which cause financial fragility and instability, contractionary PMP exacerbates the impact of bank distress on China’s financial market. In contrast, the response of SR1 initially increases but then declines following a contractionary QMP shock. While a tightening QMP may restrict the real economy in the short run, it alleviates capital misallocation and overinvestment [3,31]. Ultimately a contractionary QMP stabilizes the financial market and reduces the impact of bank distress in the long run. This is consistent with findings that China’s QMP expansion at the start of 2009 decreased MES in 2009 but significantly increased it between 2010 and 2013 [42].

The second column of Fig 7 presents the IRFs of SR2. The response of SR2 to a PMP shock is negative, indicating that tightening PMP reduces the likelihood of banks experiencing distress facing financial market turbulence. Following a contractionary PMP shock, non-state banks tend to take more risk, whereas state banks behave more cautiously [21]. As a result, SR2 for state banks decreases and increases for non-state banks, but overall SR2 across the entire banking system declines due to the dominant role of state banks. In contrast, the response of SR2 to a tightening QMP is negligible. An expansion of QMP injects liquidity into the bank system, primarily boosting loans to SOEs, which neither increases the exposure to interbank liability nor the credit risk of the bank system.

The difference between state and non-state banks may lead to different responses of systemic risks to monetary policy shocks. China’s non-state and state banks differ in their operational goal and financing channels. Compared with non-state banks, the primary goal of state banks is not to maximize profit but to implement political tasks. During the 2008 global financial crisis, even with the economic downturn and increased credit risks, the state bank followed the central government’s directive to expand its balance sheet and enhance financial support [31]. Moreover, state banks rely heavily on deposit financing. As of 2023:II, state banks held 79.93% of their liabilities as deposits, while listed non-state banks have a lower share of 65.71%.

Table 5 presents the systemic risk indicators of state banks and non-state banks. The bottom-up systemic risk indicators are lower for state banks, suggesting that a financial crisis in China is less likely to be triggered by the state banks. This could be attributed to the strong government backing of state banks, making financial distress of state banks unlikely to cause market panic. However, the top-down risk indicators of state banks are significantly higher than non-state banks. The values of SES or SRISK for state banks are nearly four times higher than those for non-state banks, indicating that the magnitude of a financial crisis in China is mainly determined by state banks. This heightened top-down systemic risk in state banks is consistent with findings that large-scale banks have high systemic importance [43].

**Table 5:** Statistics description of state and non-state banks’ systemic risks. Unit: %, billion.

		state banks		Non-state banks	
	**Indicators**	**mean**	**std**	**mean**	**std**
*Bottom-up*	CAViaR	2.12	1.13	3.14	1.33
	CoVaR	3.37	1.27	3.55	1.16
	△CoVaR	1.37	0.66	1.67	0.60
	MES	1.90	1.20	3.00	1.28
	RIS	0.59	0.41	0.69	0.29
	CATFIN	3.65	1.90	4.73	1.98
*Top-down*	SES	308.75	371.25	67.24	52.16
	SRISK	516.83	423.98	130.17	84.22

Note: Table 5 statistically describes systemic risk measures for state and non-state banks. SR1 and SR2 are PCA components derived from bottom-up and top-down risks respectively, indicating potential damage from a bank’s distress and the likelihood of encountering distress during market turbulence.

**Fig 8:**
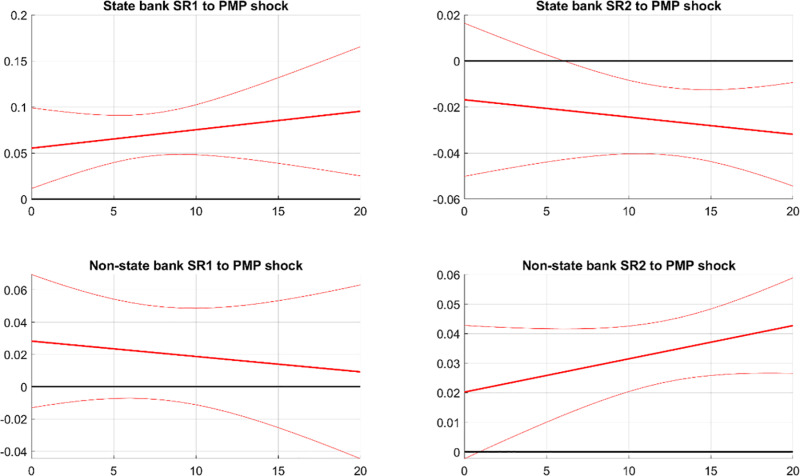
IRFs of SR in state and non-state banks to PMP. Notes: The figure illustrates the impact of contractionary PMP on systemic risks in state and non-state banks. SR1 and SR2 measure the potential damage from a bank’s distress to the financial system and the likelihood of a bank experiencing distress during market turbulence, respectively. The solid line indicates the median response, while the dashed bands show the 68% confidence interval.

Fig 8 demonstrates the effects of PMP shocks on the systemic risks of state and non-state banks. The first column shows that a contractionary PMP shock increases SR1 for both bank types, with a more pronounced effect on state banks. This suggests that tightening PMP increases potential damage from bank distress, especially for state banks. Interestingly, despite non-state banks behaving risky and state banks behaving cautiously following a contractionary PMP, SR1 mainly increases in state banks. This indicates that the risk-taking of non-state banks produces a spillover effect on state banks. The risky behavior of non-state banks results in financial fragility and instability, amplifying the adverse impacts of distress from both non-state banks and state banks. As deposit capital shifts from non-state banks to state banks following a contractionary PMP, the impact of distress of state banks is even larger than the impact of non-state banks.

The second column of Fig 8 shows the responses of SR2 to a PMP shock. After a PMP shock, SR2 decreases for state banks but increases for non-state banks. A tightened PMP widens the spread between interbank and deposit interest rates. The funding cost for non-state banks significantly increases, leading them to engage more in high-risk activities such as shadow banking to hedge the rising funding cost. In contrast with non-state banks, state banks behave more cautiously following a contractionary PMP, as they can easily increase their deposit share in liabilities to stabilize their funding cost. As a result, non-state banks become more vulnerable to market turbulence, while state banks become more robust. Thus, SR2 decreases for state banks but increases for non-state banks.

**Fig 9:**
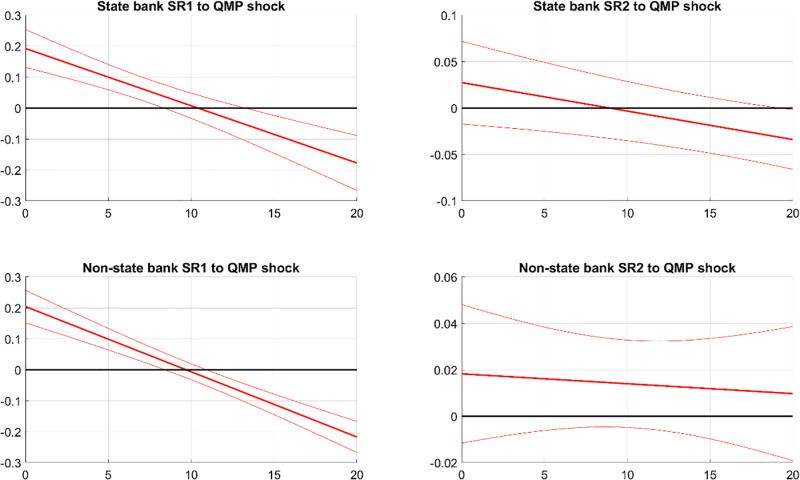
IRFs of SR in state and non-state banks to QMP shocks. Notes: The figure displays the effects of contractionary QMP on systemic risks in state and non-state banks. SR1 and SR2 respectively assess the potential damage from a bank’s distress to the financial system and the likelihood of a bank falling into distress during financial market turbulence. The solid line shows the median response, and the dashed bands represent the 68% confidence interval.

Fig 9 depicts the impact of QMP shocks on the systemic risks of state and non-state banks. The first column shows that a tightening QMP shock initially raises and then lowers SR1 for both bank types, consistent with the overall effects on the banking sector. Both state and non-state banks increase their loan to SOEs following an expansion of QMP [31], which explains why the QMP shock has a similar effect on SR1 of state banks and non-state banks.

The second column of Fig 9 reveals that a tightening QMP shock does not significantly affect the SR2 of either state or non-state banks. During a QMP expansion, banks receive liquidity from the PBC and extend more loans to SOEs, which benefit from implicit debt guarantees by the government. This expansion does not significantly alter banks’ exposure to interbank liability nor prompt them to undertake high-risk activities, suggesting that a QMP expansion is unlikely to elevate SR2 for any bank category.

**Fig 10:**
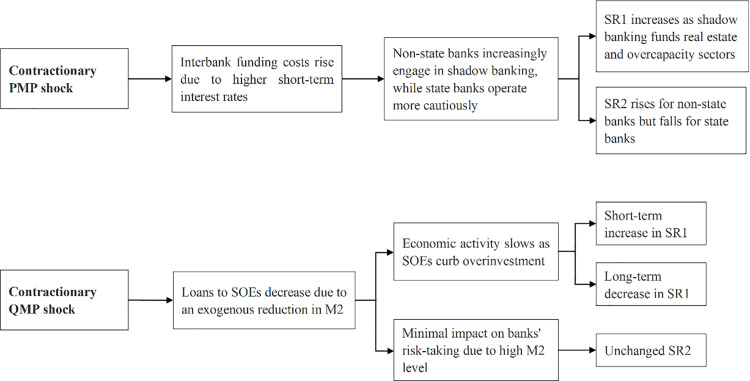
An explanation of baseline results. Notes: The figure describes how PMP and QMP shocks affect SR1 and SR2. SR1 measures the potential damage from a bank’s distress to the financial system, while SR2 measures the likelihood of a bank experiencing distress during financial market turbulence.

Fig 10 concludes the impact of China’s monetary policy shocks on banking systemic risks. When a contractionary PMP shock raises interbank interest rates, the funding costs for non-state banks increase. To maintain profitability, non-state banks increasingly engage in shadow bank activities that fund real estate and overcapacity sectors, making the financial system more vulnerable and raising SR1 for both state and non-state banks. In contrast, state banks largely avoid high-risk activities under tight monetary policy. Consequently, SR2 decreases for state banks but increases for non-state banks, with an overall decrease in SR2 due to the dominant role of state banks.

Contractionary QMP reduces M2, resulting in fewer loans to SOEs and curbing their investment. This initially raises SR1 due to reduced fixed-asset investment and slower economic activity but eventually lowers SR1 by curbing overinvestment and correcting capital misallocation between SOEs and non-SOEs. SR2 remains stable because changes in loans to SOEs have little effect on the risk-taking behavior of both state and non-state banks. Our findings are aligned with Chen et al. [21], which indicates that contractionary monetary policy leads to increased risk-taking by non-state banks. We further elucidate that it is more likely the contractionary PMP, rather than QMP, that encourages risk-taking by non-state banks. In addition, the risk-taking behavior of non-state banks might produce a spillover effect, amplifying the damage of financial distress for both state and non-state banks.

### 3.5. The effects of bank size and funding structure

When considering the contribution to bank systemic risks, the bank size and funding structure are the dominant factors. Bank systemic risks tend to grow with a larger bank size [44–47] and with a higher ratio of short-term liability such as short-term wholesale funding [48,49]. Given the importance of bank size and funding structure in determining bank systemic risks, we examine how they affect the response of bank systemic risks to monetary policy.

**Fig 11:**
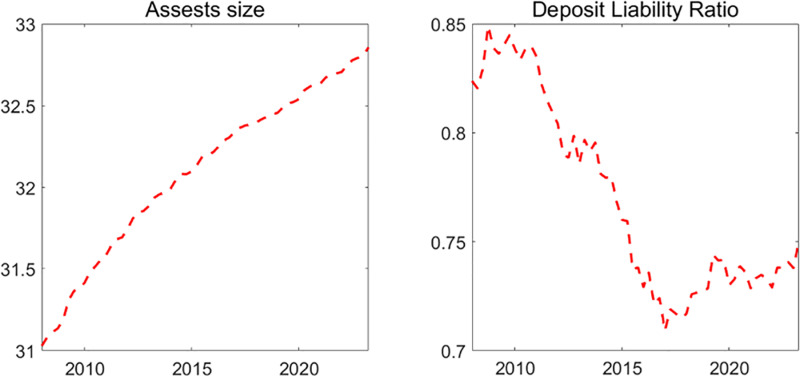
Bank Size and Deposit Liability Ratio. Notes: Asset size is defined as the logarithm of the total assets of banks. The Deposit Liability Ratio is the proportion of deposits to total liabilities. Data is from CEIC.

Fig 11 showcases China’s bank size and deposit liability ratio from 2007 to 2023, derived from aggregated data of publicly listed banks. The asset size is represented as the logarithm of the total bank assets. The Deposit liability ratio is the proportion of total deposits to total bank liabilities. The left panel highlights a consistent growth in the assets of Chinese banks, with the asset size in 2023:I being 6.24 times that of 2007:IV. The right panel charts the fluctuations in the deposit liability ratio, which witnessed a decrease of approximately 7.36% in 2023:I compared to 2007: I.

To assess the role of bank size and funding structure, we include an interaction term in the baseline model to reexamine how China’s monetary policy affects its banking systemic risks. The extended model can be presented as follows:


$$\begin{array}{c} \begin{array}{c} SR_{t+h}=\alpha_{h}+\beta_{h}MP_{t}+\theta_{h}Ind_{t}\times MP_{t}+\gamma_{1}SR_{t-1}+\gamma_{2}SR_{t-2}\\ +\eta_{1,\;h}{GDP}_{t}+\eta_{2,\;h}{GDP}_{t-1}+\eta_{3,\;h}{GDP}_{t-2}+u_{t+h} \end{array} \end{array}$$ (6)

where *h* is prediction periods ranging from 0 to 20, *SR* denotes SR1 or SR2, *MP* is PMP or QMP shocks, *Ind* is the logarithm of either total bank assets or deposit liability ratio, GDP is GDP real growth rate, *u* is the prediction error. We shrink the estimated IR toward a line by setting the estimated multiplier *β*_*h*_ to a linear polynomial, which is roughly consistent with the IR estimated by the standard LP.

#### 3.5.1. Bank size.

**Fig 12:**
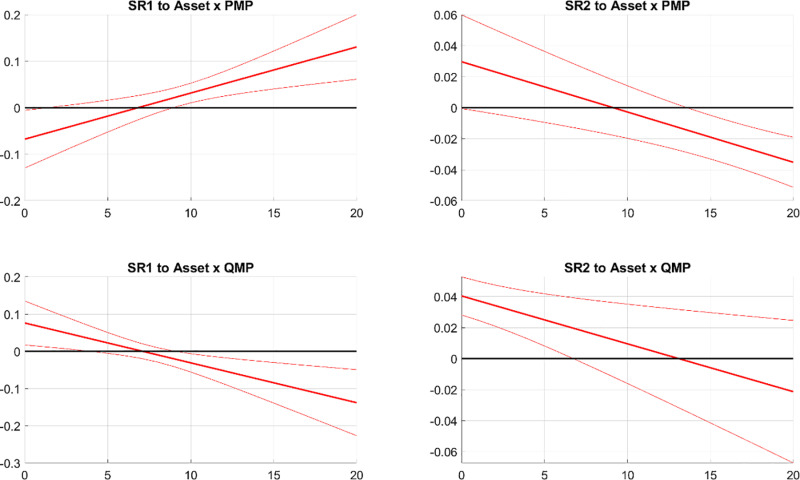
The responses of SR to monetary policy shocks and asset size interaction. Notes: The figure displays the effects of the interaction between asset size and monetary policy shocks on systemic risks. SR1 and SR2 respectively indicate the potential damage from a bank’s distress to the financial system and the likelihood of a bank experiencing distress during financial market turbulence. The solid line shows the median response, and the dashed bands represent the 68% confidence interval.

Fig 12 illustrates the responses of banks’ systemic risks to the interaction term of banks’ asset size and monetary policy shocks, denoted as Assets × PMP shock and Assets × QMP shock. In the first column, the response of SR1 is positive to Assets × PMP shock and turns from positive to negative in response to Assets × QMP shock. This aligns with the reactions of SR1 to PMP and QMP shocks, indicating that the response of SR1 to monetary policy shocks intensifies with the size of the bank assets. In the second column of Fig 12, the response of SR2 to Assets × PMP shock or Assets × QMP shock is barely significant. This suggests that the size of bank assets has very little effect on how PMP and QMP affect SR2.

#### 3.5.2. Funding structure.

**Fig 13:**
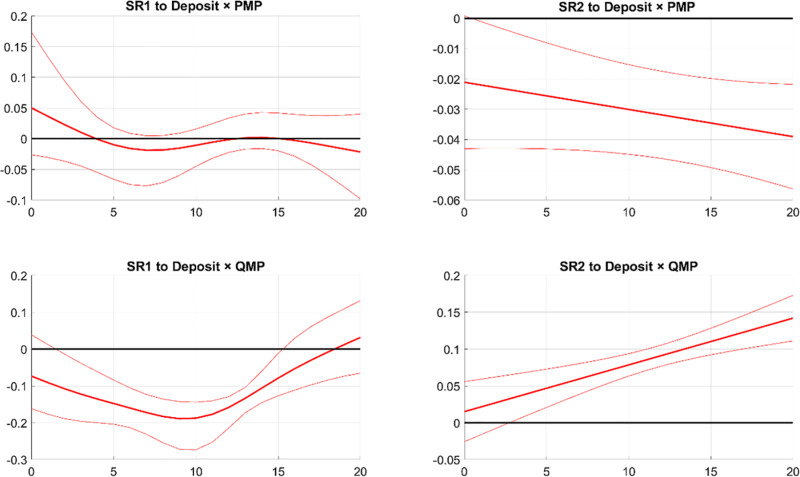
The responses of SR to monetary policy shocks and deposit ratio interaction. Notes: The figure depicts the effects of the interaction between the deposit liability ratio and monetary policy shocks on systemic risks. SR1 and SR2 measure the potential damage from a bank’s distress to the financial system and the likelihood of a bank experiencing distress during financial market turbulence, respectively. The solid line shows the median response, and the dashed bands represent the 68% confidence interval.

Fig 13 illustrates the responses of banks’ systemic risks to the interaction term of deposit liability ratio and monetary policy shocks, denoted as Deposit× PMP shock and Deposit× QMP shock. In the first column, the response of SR1 is insignificant to Deposit × PMP shock and significantly negative to Deposit × QMP shock. This implies that a higher deposit-debt ratio amplifies the negative effects of contractionary QMP on SR1. In the second column of Fig 13, SR2 reacts negatively to Deposit × PMP shock. This indicates that a higher deposit-debt ratio intensifies the negative effects of tightened PMP on SR2. The response of SR2 to Deposit × QMP shock is significantly positive, indicating that tightened QMP would weaken the resilience of banks that rely more on deposit financing.

## 4. Robustness checks

**Fig 14:**
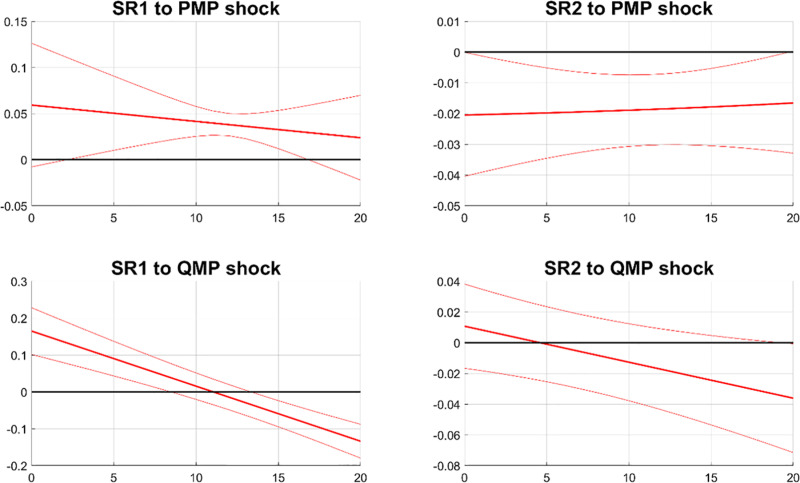
IRFs of banks’ systemic risks when using 4 lags.

We conduct two robustness checks to evaluate the reliability of our empirical results. First, we adjust the lag length of the baseline model from 2 to 4. Fig 14 presents the results, which remain qualitatively consistent with the baseline results. This suggests that altering the lag order does not significantly change the baseline results.

**Fig 15:**
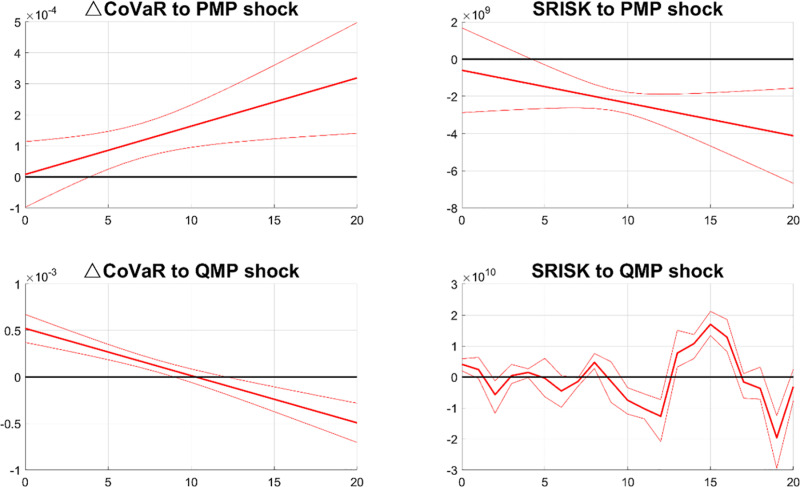
IRFs of △CoVaR and SRISK. Notes: The figure shows the effects of contractionary PMP and QMP shocks on the systemic risks of the bank.

Second, instead of using PCA measures, we directly employ △CoVaR and SRISK to measure bottom-up and top-down systemic risks. SRISK is first-order differenced to obtain a stable series. The empirical results are shown in Fig 15. Consistent with the baseline results, contractionary PMP shocks raise △CoVaR but mainly decrease SRISK, while tightening QMP shocks initially increase then decrease △CoVaR, and have no significant effect on SRISK.

△CoVaR and SRISK respectively reflect the potential damage of a bank’s distress to the entire financial system and the likelihood of a bank falling into distress during financial market turbulence. The solid line represents the median response and the dashed bands display the 68% confidence interval.

**Fig 16:**
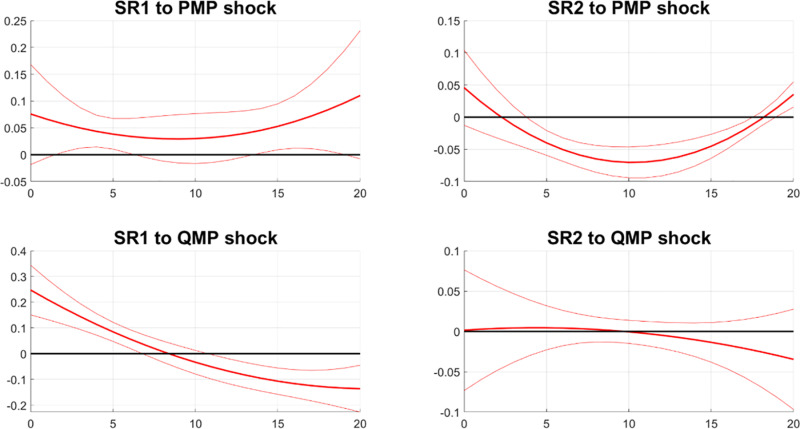
IRFs of banks’ systemic risks when *β*_*h*_** shrinking to order 2 polynomial.** Notes: The figure illustrates the responses of SR1 and SR2 to PMP and QMP shocks. the estimated multiplier βh shrinks toward a polynomial of order 2. SR1 and SR2 measure the potential damage from a bank’s distress to the financial system and the likelihood of a bank experiencing distress during financial market turbulence, respectively. The solid line shows the median response, while the dashed bands represent the 68% confidence interval.

Finally, we retested the empirical results after shrinking the estimated multiplier *β*_*h*_ toward a polynomial of order 2. The empirical results are displayed in Fig 16. Consistent with the baseline results, the contractionary PMP shock raises SR1 but mainly decreases SR2, while tightening QMP shocks initially increase and then decrease SR1 but have no significant effect on SR2.

## 5. Conclusion and Policy Implication

This paper examines the differential impacts of PMP and QMP on banking systemic risks in China. We compute seven systemic risk indicators and amalgamate them into bottom-up and top-down systemic risk indicators. Using an SVAR with four variables, we identify the PMP and QMP and then apply SLP models to investigate the impacts of PMP and QMP shocks on both bottom-up and top-down systemic risks. The results show that contractionary PMP shocks intensify the bottom-up banking systemic risks while alleviating the top-down ones. Conversely, contractionary QMP shocks initially amplify the bottom-up systemic risk, which then gradually subsides, with minimal impact on the top-down systemic risk. When distinguishing between state and non-state banks, the effect of PMP on systemic risks of the banking sector significantly changed while the effect of QMP remains consistent. Tightening PMP increases bottom-up systemic risks for both state and non-state banks, with a larger effect on state banks; tightening PMP reduces top-down systemic risk in state banks, but exacerbates it in non-state banks. Finally, characteristics of the banks, including asset size and the extent of deposit reliance, are significant factors in determining the effect of monetary policy on banking systemic risks.

Three key policy implications emerge from our findings. First, contractionary PMP should be avoided as it increases the vulnerability of non-state banks and the potential impact of banking distress. Second, an expansionary PMP increases the systemic importance of state banks. Thus, when implementing expansionary PMP, the PBC should adopt necessary measures to mitigate the “too big to fail” issue among state banks. Lastly, the PBC should not rely on expansionary QMP to manage systemic risks under normal conditions. While expanding QMP may provide short-term stability, it exacerbates systemic risks over time.

The limitation of our research. Firstly, we do not discuss whether monetary policy has asymmetric effects on systemic risks. Secondly, the effects of PMP and QMP on systemic risks may vary in different business cycles, which is rarely discussed in this study. Finally, we focus on the aggregate effect of PMP and QMP on systemic risks, but it is also important to study the effect of PMP and QMP on systemic risks from a micro perspective.

## Supporting information

S1 Data Systemic risk indicators.
(RAR)

S2 Data Identification Scheme. (DOCX)
