# Supplementary material for: Divergent impacts of quantity versus price-based monetary policies on banking systemic risk: Evidence from China
Source: PLoS One. 2025 May 28;20(5):e0322709. doi: 10.1371/journal.pone.0322709 (PMC12118993; doi:10.1371/journal.pone.0322709)
Supplement: S2 Data — (DOCX) [file pone.0322709.s002.docx]

Following B&H, our identification scheme consists of three parts. First, we specify the structural equations. Second, we define the prior densities of contemporaneous structural parameters. Finally, we supplement the identification with several sign restrictions.

The structural equations consist of the Phillips curve, the IS curve, the QMP, and PMP monetary policy equations. The following equation (1) gives the Phillips curve,

$$\begin{aligned} \pi_{t}=c^{s}+\lambda y_{t}+{\beta E}_{t}\pi_{t+1}+u_{t}^{s}\#\left( 1 \right) \end{aligned}$$

Where $\pi_{t}$ and $E_{t}\pi_{t+1}$ are inflation and its one-step-ahead forecast value,$y_{t}$ is output,$\lambda$ and $\beta$ capture the effect of output and expected inflation on the inflation,$c^{s}$ is the constant,$u_{t}^{s}$ is the supply shock.

The intertemporal IS curve is described as (2)

$$\begin{aligned} y_{t}=c^{d}+\xi E_{t}y_{t+1}-\kappa\left( i_{t}-E_{t}\pi_{t+1} \right)+\vartheta m_{t}+u_{t}^{d}.\#\left( 2 \right) \end{aligned}$$

Where $E_{t}y_{t+1}$ is one-step-ahead forecast output, $i_{t}$ is interest rate and $m_{t}$ denotes money growth rate, respectively. $\xi$ measures the effect of forward-looking output; $\kappa$ depicts the effect of interest rate, and $\vartheta$ represents the effect of money growth rate;$c^{d}$ is constant and $u_{t}^{d}$ denotes the demand shock.

Following B&H, we let $E_{t}y_{t+1}=a_{y}y_{t}$, $E_{t}\pi_{t+1}=a_{\pi}\pi_{t}$, and the prior expectation is $a_{y}=a_{\pi}=0.75$. Then the equations (1) and (2) can be written as $\begin{aligned} y_{t}=k^{s}+\alpha^{s}\pi_{t}+\tilde{u}_{t}^{s},\#\left( 3 \right) \end{aligned}$

Where $k^{s}=-\frac{c^{s}}{\lambda}$,$\alpha^{s}=\frac{1-\beta a_{\pi}}{\lambda}$,$\tilde{u}_{t}^{s}=-\frac{u_{t}^{s}}{\lambda}$, and

$$\begin{aligned} y_{t}=k^{d}+\beta^{d}\pi_{t}+\beta^{r}i_{t}+\beta^{m}m_{t}+\tilde{u}_{t}^{d},\#\left( 4 \right) \end{aligned}$$

Where $k^{d}=\frac{k^{d}}{(1-a_{y}\xi)}$，$\beta^{r}=-\frac{\kappa}{(1-a_{y}\xi)}$,$\beta^{d}=\frac{a_{\pi}\kappa}{(1-a_{y}\xi)}$，$\beta^{m}=\frac{\vartheta}{1-a_{y}\xi}$，$\tilde{u}_{t}^{d}=\frac{1}{(1-a_{y}\xi)}u_{t}^{d}$.

For PMP and QMP equations, we set them as the following two equations,

$$\begin{aligned} i_{t}=c^{i}+\rho_{r}i_{t-1}+\left( 1-\rho_{r} \right)\psi^{\pi}\pi_{t}+\left( 1-\rho_{r} \right)\psi^{y}y_{t}+\varepsilon_{t}^{r}\#\left( 5 \right) \end{aligned}$$

$$\begin{aligned} m_{t}=c^{m}+\rho_{m}m_{t-1}+\left( 1-\rho_{m} \right)\gamma^{\pi}\pi_{t}+\left( 1-\rho_{m} \right)\gamma^{y}y_{t}+\varepsilon_{t}^{m}.\#\left( 6 \right) \end{aligned}$$

where $c^{i}$ and $c^{m}$ are constants, $\rho_{r}$ and $\rho_{m}$ are smooth parameters, $\psi^{\pi}$ and $\psi^{y}$ describe the responses of the policy rate to the variation of output and inflation. $\gamma^{\pi}$ and $\gamma^{y}$ depict the responses of the money supply to the variation of output and inflation. $\varepsilon_{t}^{r}$ and $\varepsilon_{t}^{m}$ are PMP and QMP shocks, respectively.

Next, We define the prior for the contemporaneous structural parameter. In equation (3), $\alpha^{s}=\frac{1-\beta a_{\pi}}{\lambda}$ , where the value of $\lambda$ is a function of the intertemporal elasticity of substitution$\sigma$, and the reciprocal of labor supply elasticity $\eta$, the price rigidities extent $\theta$, and the discount rate $\beta$, i.e., $\lambda=\frac{(\sigma+\eta)\left( 1-\theta\beta\right)\left( 1-\theta\right)}{\theta}$. Research in China usually set $\beta=$0.98 and $\theta$=0.75, $\sigma$ and $\eta$ lie in the interval between 0 and 3. We set $\beta=$0.98，$\theta$=0.75，$\eta=\sigma=2$ and thus $\lambda=0.35$, $\alpha^{s}=$0.75. In equation (4), $\sigma=2$ indicates its reciprocal $\kappa=0.5$, $\xi$ often takes the value between 0.37 and 1(Benati,2008). $\vartheta$ is 0.06 in Liu(2010) and 0.05 in Wu and Lian(2015). We set $\xi=$0.5，$\kappa$=0.5，$\vartheta=0.05$, and thus $\beta^{r}=-0.8$，$\beta^{d}=0.6$，$\beta^{m}=0.11$.

We mainly refer to China's domestic researches to set the prior mode for the QMP and PMP equations' structural parameters. Table 1 summarizes the existing estimates for the structural parameters in PMP and QMP equations from relevant studies.^[[1]](#endnote-1)^ These estimates reflect differences in model specification, sample period, variable. We average these estimates to get the prior parameters of PMP and QMP equations.

**Table 1**. The estimates of parameters in PMP and QMP

|  | PMP | | | QMP | | |
| --- | --- | --- | --- | --- | --- | --- |
|  | $\rho_{r}$ | $\psi^{\pi}$ | $\psi^{y}$ | $\rho_{m}$ | $\gamma^{\pi}$ | $\gamma^{y}$ |
| Xie and Luo(2004) | 0.81 | 2.84 | 0.81 |  |  |  |
| Jiang and Chu(2014) | 0.92 | 0.97 | 1.5 |  |  |  |
| Chen et al.(2016) | 0.87 | 1.7 | 0.55 |  |  |  |
| Wang et al.(2016) | 0.49 | 1.6 | 0.3 |  |  |  |
| Liu and Zhang(2010) | 0.82 | 0.83 | 2.83 | 0.88 | -0.50 | -1.33 |
| Yue and Niu(2014) | 0.95 | 1.00 | 0.25 | 0.70 | -1.63 | -0.21 |
| Wu and Lian(2016) | 0.64 | 1.08 | 0.08 | 0.90 | -1.00 | -1.90 |
| Zhuang et al.(2017) | 0.43 | 3.00 | 0.70 | 0.65 | -1.00 | -0.63 |
| Mean | 0.75 | 1.63 | 0.88 | 0.78 | -1.03 | -1.02 |

Table 2 summarizes the prior densities for contemporaneous structural parameters. Following B&H, for prior densities of the parameters excepting $\rho_{r}$ or $\rho_{m}$, We use a student $t$ distribution with scale parameter 0.4, and 3 degrees of freedom; for prior densities of $\rho_{r}$ or $\rho_{m}$, We use a beta distribution with standard deviation 0.2. We also restrict $\beta^{r}\leq0$ and $\beta^{m}\geq0$, because the decrease of interest rate or increase in money supply is not supposed to stimulate the aggregate demand. We additionally restrict $\psi^{y}, \psi^{\pi}\geq0$ and $\gamma^{y},\gamma^{\pi}\leq0$ since China’s central bank (People’s Bank of China, PBC) reacts to the variation of output or inflation counter-cyclically. We do not restrict the range of $\alpha^{s}$ and $\beta^{d}$ since we do not have a strong prior belief about their signs.

**Table 2.** Priors for contemporaneous coefficients

| Parameter | Implication | Range | Prior mode | Prior scale |
| --- | --- | --- | --- | --- |
| Student $t$ distribution with 3 degrees of freedom | | | | |
| $\alpha^{s}$ | Effect of $\pi$ on supply | arbitrary | 0.75 | 0.4 |
| $\beta^{d}$ | Effect of $\pi$ on demand | arbitrary | 0.6 | 0.4 |
| $\beta^{r}$ | Effect of $i$ on demand | ≤0 | -0.8 | 0.4 |
| $\beta^{m}$ | Effect of $m$ on demand | ≥0 | 0.11 | 0.4 |
| $\psi^{y}$ | Response of $i$ to $y$ | ≥0 | 0.88 | 0.4 |
| $\psi^{\pi}$ | Response of $i$ to $\pi$ | ≥0 | 1.63 | 0.4 |
| $\gamma^{y}$ | Response of $i$ to $y$ | ≤0 | -1.02 | 0.4 |
| $\gamma^{\pi}$ | Response of $i$ to $\pi$ | ≤0 | -1.03 | 0.4 |
| *Beta distribution with α=*3.05 and *β*=1.68 | | | | |
| $\rho_{r}$ | Interest rate smoothing | [0,1] | 0.75 | 0.2 |
| $\rho_{m}$ | Money supply smoothing | [0,1] | 0.75 | 0.2 |

Finally, we write the contemporaneous coefficients matrix $A_{base}$as (7), and supplement the identification with some reasonable sign restrictions by constraining the inverse of $A_{base}$. Three sign restrictions are added as follows:

$\boldsymbol{H}_{\boldsymbol{1}}$: a positive supply shock increases the contemporaneous output.

$\boldsymbol{H}_{\boldsymbol{2}}$: a contractionary PMP shock raising the interest rate has a contemporaneous negative impact on output.

$\boldsymbol{H}_{\mathbf{3}}:$a contractionary QMP shock decreasing the money supply has a contemporaneous negative impact on output.

The equations from (7) to (9) below summarize the sign restrictions, where $h_{1}$,$h_{2}$, and $h_{3}$ are subject to the asymmetric Student t distribution proposed by B&H(2019).^[[2]](#endnote-2)^ For each sign restriction, we also allow a 7.1% chance that the restriction is invalid.

$$\begin{aligned} A_{base}=\left[ \begin{matrix} 1 & -\alpha^{s} & 0 & 0 \\ 1 & -\beta^{d} & -\beta^{r} & -\beta^{m} \\ -\left( 1-\rho_{r} \right)\psi^{y} & -\left( 1-\rho_{r} \right)\psi^{r} & 1 & 0 \\ -\left( 1-\rho_{m} \right)\gamma^{y} & -\left( 1-\rho_{m} \right)\gamma^{\pi} & 0 & 1 \end{matrix} \right]\#(7) \end{aligned}$$

$$\begin{aligned} p(h_{1}>0)=p(A_{base}^{-1}\left( 1,1 \right)>0)=92.9\%\#\left( 8 \right) \end{aligned}$$

$$\begin{aligned} p(h_{2}<0)=p(A_{base}^{-1}\left( 1,3 \right)/A_{base}^{-1}\left( 3,3 \right)<0)=92.9\%\#\left( 9 \right) \end{aligned}$$

$$\begin{aligned} p(h_{3}>0)=p(A_{base}^{-1}\left( 1,4 \right)/A_{base}^{-1}\left( 4,4 \right)>0)=92.9\%.\#\left( 10 \right) \end{aligned}$$

1. 1. For coefficients corresponding to $\rho_{m}\gamma^{\pi}$ or $\rho_{m}\gamma^{y}$, we divide these coefficients by the $\rho_{m}$ to get $\gamma^{\pi}$ or $\gamma^{y}$.

   [↑](#endnote-ref-1)
2. 1. In this paper, the location parameter equals 0 and the scale parameter equals 1.

   # References

   Xie, P. & Luo, X., 2004, "Taylor Rule and Its Empirical Test in China' s Monetary Policy", *Economic Research Journal,* No. 3, pp. 3-12.

   Jiang, H. & Chu, Z., 2014, "Aggregate Supply,Effect of Monetary Policy, Adaptive Learning Expectation, And Effectiveness of Monetary Policy", *Journal of Financial Research,* Vol. No.407, No. 5, pp. 5-20.

   Chen, C.-L., Zheng , T.-G. & Yao, S., 2016, "A Time-varying Parameter Taylor Rule and the Central Bank’s Monetary Policy Orientation", *Economic Research Journal,* Vol. 051, No. 008, pp. 43-56.

   Wang, X., Wang, Q. & Chen, Z.-F., 2016, "Monetary Policy Expectation and Inflation Management: A DSGE Analysis Based on News Shocks", *Economic Research Journal,* No. 2, pp. 16-29.

   Liu, L.-G. & Zhang, W., 2010, "A New Keynesian model for analysing monetary policy in Mainland China", *Journal of Asian Economics,* Vol. 21, No. 6, pp. 540-51.

   Yue, C. & Niu, L., 2014, "Estimating and Comparing China's Monetary Policy Rules within a DSGE Model", *The Journal of Quantitative & Technical Economics,* No. 3, pp. 119-33.

   Wu, G. & Lian, F., 2016, "Research on the transmission of China's Monetary policy: An explosion of quantity and price hybrid rule ", *The Journal of World Economy,* Vol. 39, No. 3, pp. 3-25.

   Zhuang, Z., Jia, H. & Liu, D.-M., 2018, "A Study on the Macroeconomic Effects of Monetary Policy：Perspective of Anticipated and Unanticipated Shocks", *China Industrial Economics,* Vol. No.364, No. 07, pp. 80-97. [↑](#endnote-ref-2)
